# Roles of host mitochondria in the development of COVID-19 pathology: Could mitochondria be a potential therapeutic target?

**DOI:** 10.1186/s43556-021-00060-1

**Published:** 2021-11-23

**Authors:** Kavya Srinivasan, Ashutosh Kumar Pandey, Ashlena Livingston, Sundararajan Venkatesh

**Affiliations:** 1 Department of Microbiology, Biochemistry and Molecular Genetics, Rutgers -New Jersey Medical School, The State University of New Jersey, Newark, NJ USA; 2 New York Institute of Technology, Old Westbury, NY USA; 3 Department of Pharmacology, Physiology and Neuroscience, Rutgers -New Jersey Medical School, The State University of New Jersey, Newark, NJ USA; 4 Dallas Baptist University, Dallas, TX United States

**Keywords:** COVID-19, SARS-CoV-2, Mitochondria, ACE-2 receptor, Cytokine storm, Inflammation

## Abstract

The recent emergence of severe acute respiratory syndrome-Corona Virus 2 (SARS-CoV-2) in late 2019 and its spread worldwide caused an acute pandemic of Coronavirus disease 19 (COVID-19). Since then, COVID-19 has been under intense scrutiny as its outbreak led to significant changes in healthcare, social activities, and economic settings worldwide. Although angiotensin-converting enzyme-2 (ACE-2) receptor is shown to be the primary port of SARS-CoV-2 entry in cells, the mechanisms behind the establishment and pathologies of COVID-19 are poorly understood. As recent studies have shown that host mitochondria play an essential role in virus-mediated innate immune response, pathologies, and infection, in this review, we will discuss in detail the entry and progression of SARS-CoV-2 and how mitochondria could play roles in COVID-19 disease. We will also review the potential interactions between SARS-CoV-2 and mitochondria and discuss possible treatments, including whether mitochondria as a potential therapeutic target in COVID-19. Understanding SARS-CoV-2 and mitochondrial interactions mediated virus establishment, inflammation, and other consequences may provide a unique mechanism and conceptual advancement in finding a novel treatment for COVID-19.

## Introduction

COVID-19 is caused by a new coronavirus, identified as a severe acute respiratory syndrome- coronavirus 2 (SARS-CoV-2), stirring the globe since the start of its outbreak in late 2019. At the time of the final submission of this article on 20^th^ October 2021, around ~246 million people had been infected with SARS-CoV-2 and close to 5 million had died, which is approximately a 2% death rate across the globe [1]. Although the zoonotic origin of the virus is still not clear, it is believed that most likely the origin is related to bat SARS-related CoV, based on the genomic sequence similarity [2]. Until the recent rollout of the vaccines, the only possible prevention methods have been a combination of face masking, social distancing, and improved personal hygiene. Rapid advancement in SARS-CoV-2 vaccine research, emergency and fast-track approvals of various COVID-19 vaccines prevented casualties and mortalities at a high rate [3]. However, as cases continue to rise with the emergence of new variants (e.g., delta, epsilon, etc.), there is an unmet need for understanding the mechanisms of COVID-19 pathologies for diagnosis, prevention, and treatment [4]. Although COVID-19 vaccines are shown to prevent severe illness and hospitalization, the long-term effect of this virus and various forms of its variants on human health are not known as COVID-19 breakthrough has also been reported in vaccinated groups as well [5–7]. However, the symptoms in these groups are mostly mild or none [7]. Therefore, understanding SARS-CoV-2’s mechanisms of host interaction and disease establishment is important to develop a novel therapeutic tool to combat this highly contagious disease.

Angiotensin-converting enzyme-2 (ACE-2) receptor-mediated entry of the virus is considered as the primary infection mechanism; however, the consequence after entry and mechanisms of pathologies leading to mortality, cytokine storm, and inflammation are not well known. Furthermore, many newfound studies suggest the potential involvement of host mitochondria in COVID-19 infection is believed to be one of the key mechanisms for COVID-19 pathologies [8–11]. Therefore, in this review, we seek more clarity surrounding mitochondria and their role in establishing COVID-19 infection and explore their possible role as a potential therapeutic target in COVID-19 treatment.

## SARS-CoV-2 Structure

**Fig. 1:**
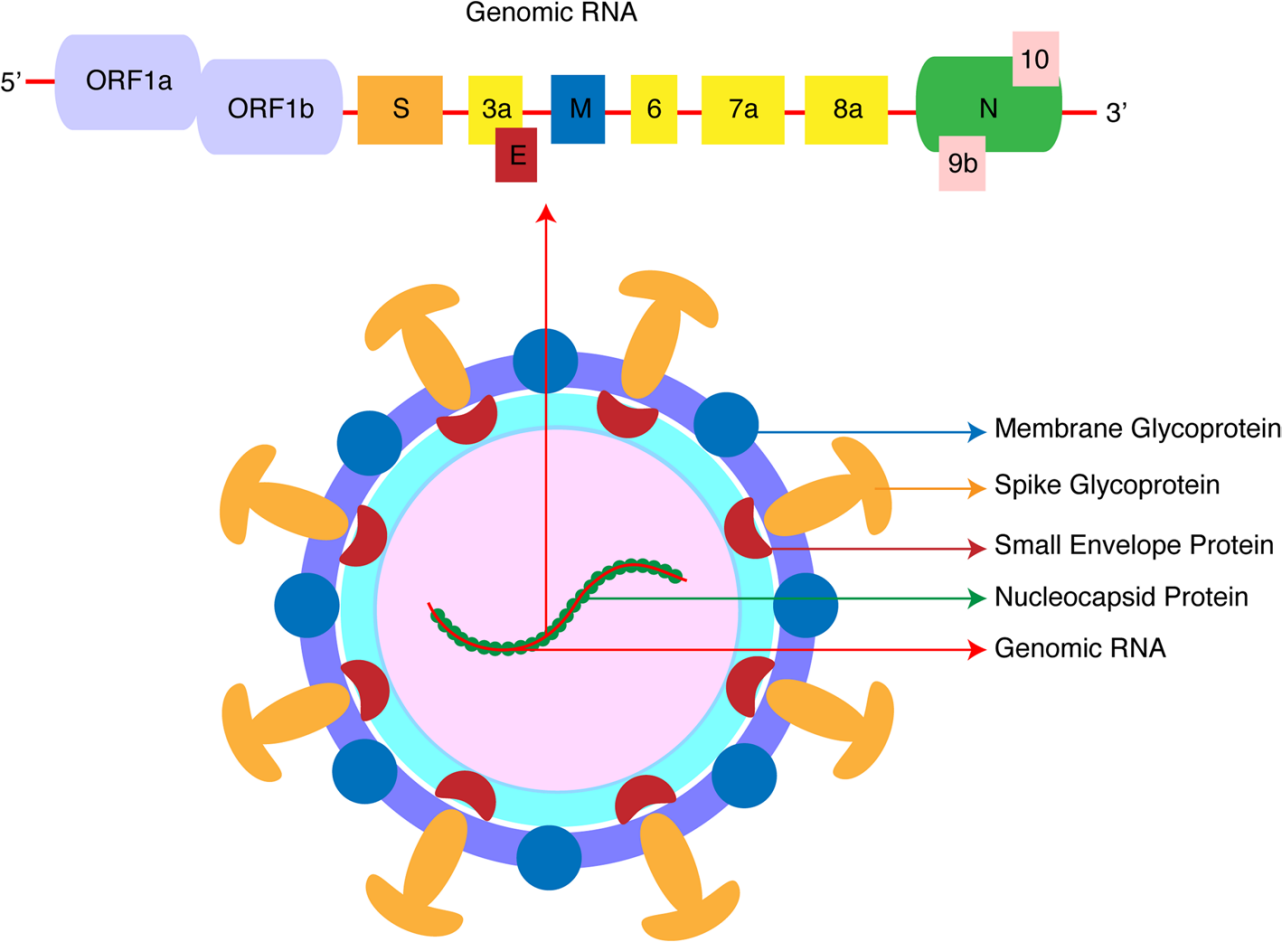
Schematic representation of the structure of SARS-CoV-2 virus. SARS-CoV-2 is a RNA virus primarily made up of spike (S), membrane (M), envelope (E), and nucleocapsid (N) structural proteins embedded in the lipid bilayer of the viral envelope. The N glycoprotein is associated with the virion's genomic RNA, which is approximately 30 kb. In addition, SARS-CoV-2 consists of non-structural and other open reading frames (ORFs) that are believed to help the virus in establishing its infection and various steps of COVID-19 disease progression

Coronaviruses belong to the family of *coronaviridae* and order *Nidovirales,* contain a large single-stranded RNA, and have a lipid envelope with spike proteins. When observed under the electron microscope, coronavirus appears as a crown-like structure due to their spike glycoproteins [12] (Fig. 1). Like most notable human disease-causing viruses, SARS-CoV-2 also contains a positive-sense single-stranded RNA, belonging to a subgroup of the coronavirus family known as beta-coronavirus. Other notable RNA viruses are those that cause the common cold, dengue, Ebola, hepatitis C, hepatitis E, influenza, measles, MERS, polio, rabies, SARS, and West Nile fever. Among these, SARS-CoV-2 is more contagious than its counterparts, transferred to humans at an exceptionally high infection rate that it has been estimated that the transmissibility rate (*R*_0_) for SARS-CoV-2 to be in the average of 2.5 compared to 2.4 for SARS-CoV, 2.0 for the 1918 influenza pandemic, 1.7 for the 2009 influenza pandemic and 0.9 for MERS-CoV [13]. From so far identified coronaviruses, HCoV 229E, HKU1, NL63, and OC43 cause mild upper respiratory tract infections and are responsible for most of the common cold [14]. In contrast, SARS-CoV, MERS-CoV, and the recently identified SARS-CoV-2 are highly infectious and cause severe life-threatening diseases [14–19].

The SARS-CoV-2’s RNA genome is about 29.9 kb long and encodes four structural proteins (S, E, M, and N) (Fig. 1) [20]. The structural proteins are surface spike glycoprotein (S), the envelope glycoprotein protein (E), membrane glycoprotein (M), and nucleocapsid protein (N) (Fig. 1) [21, 22]. SARS-CoV-2’s RNA genome was also shown to encode sixteen non-structural proteins (nsp1−16). In addition, many open reading frames (ORFs) are being identified and reported. Although ORFs are not essential for viral replication but are believed to be necessary for viral pathogenesis. It has been predicted and reported several ORFs (3a, 3b, 6, 7a, 7b, 8, 9b, 9c, 10) in SARS-CoV-2 [23, 24].

Like SARS-CoV, SARS- CoV-2 also infects the host cell using the 150 kDa ‘S’ spike protein [25, 26]. However, ‘N’ glycoprotein is the most abundant structural protein, conserved among other coronaviruses, associated with the viral genome, and forms a ribonucleoprotein core of the virus [27]. While the coronavirus ‘M’ protein is involved in determining the virus's structure and shape, including stabilizing the N protein-RNA complex, the ‘E’ structural protein, which is the smallest of all structural proteins, helps in the production and maturation of the virus [28, 29]. Although ACE-2 is widely expressed in many organs and tissues, the respiratory tract cells remain the major port of entry. The ACE2 receptor, through which SARS-CoV-2 binds and enters the host cells, is a carboxypeptidase that functions as a counterbalance to the angiotensin-converting enzyme (ACE) of the renin-angiotensin-aldosterone system (RAAS) by converting angiotensin II to angiotensin (1-7). As a result, cells expressing ACE-2 are at a higher risk of establishing infection. SARS-CoV-2 has Open Reading Frames (ORFs) responsible for various steps of its disease progression, where ORF1ab is responsible for most enzymatic proteins, including S, E, M, and N [30].

## SARS-CoV-2 host cell entry mechanisms and its affinity to various tissues

**Fig. 2:**
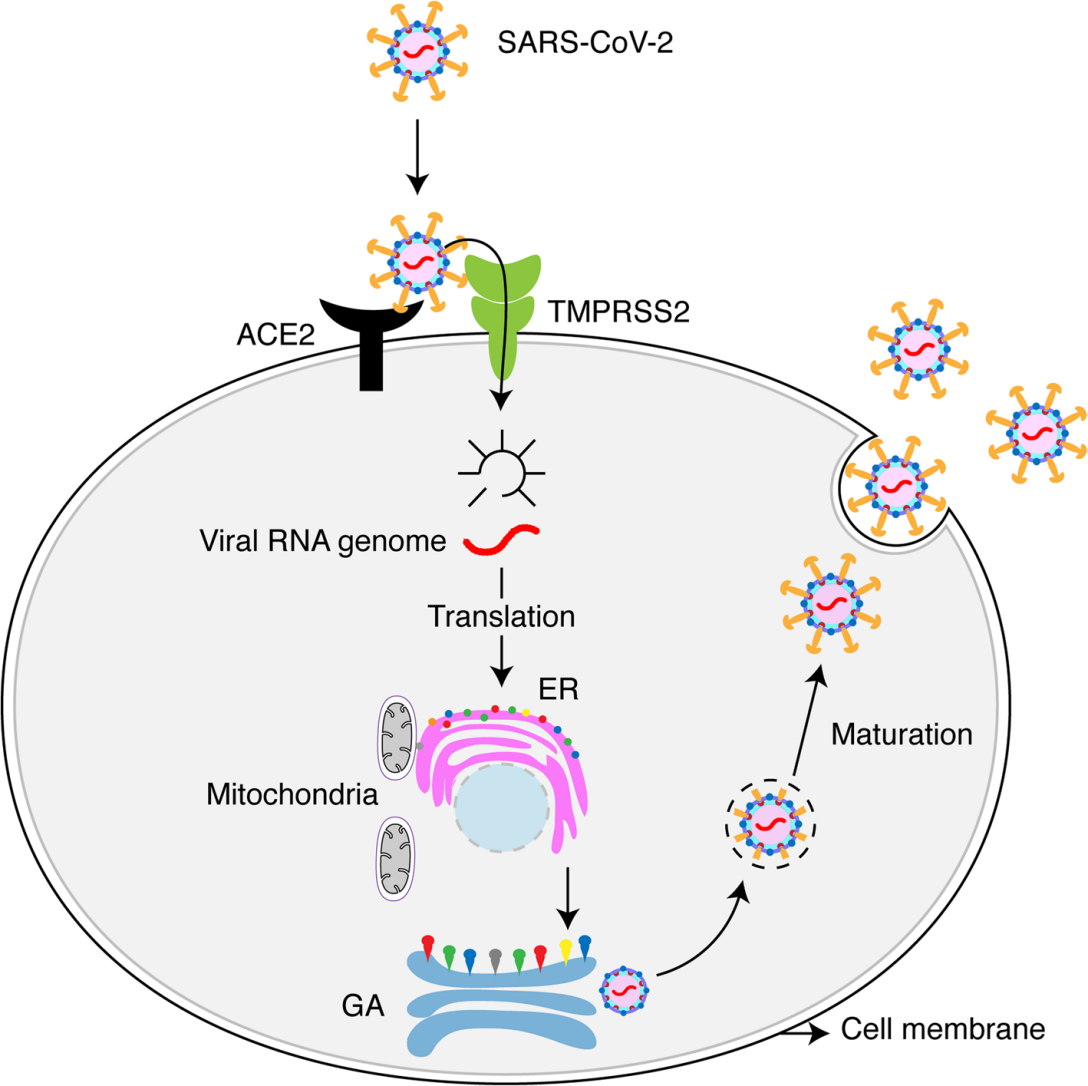
Schematic representation of SARS-CoV-2 entry and replication in host cells. SARS-CoV-2 enters host cells through its spike ‘S’ protein by interacting with the host’s ACE-2 receptor, where S is primed by the host cell serine protease, TMPRSS2. Upon entry, the release of viral genomic RNA subsequently leads to genomic replication using the host cell’s translational and transcriptional machinery. Structural proteins are synthesized and assembled at the Endoplasmic reticulum (ER) and Golgi apparatus (GA), respectively, following their maturation and release

One of the significant mechanisms of coronavirus entry known to date is that the virus uses the ‘S’ protein and the host’s ACE-2 receptor to facilitate its access into the host cell. In contrast, S protein is primed by the host cell serine protease, transmembrane protease, serine 2 (TMPRSS2) [31, 32] (Fig. 2). Importantly, SARS-CoV-2 spike protein ‘S’ binds human ACE2 receptors through its receptor-binding domain (RBD), which then subsequently proteolytically primed by human proteases to enter the cell [25]. However, furin, a human protease (convertase) predominantly preactivates SARS-CoV-2 entry, reducing the virus dependency on human target cell proteases [25]. Interestingly, SARS-CoV-2 RBD, which has a high affinity to ACE2, also possesses hidden RBD to evade innate immune responses [25].

The ACE-2 receptor is commonly expressed in the lungs, intestine, kidneys, and heart [33–36]. Compared to the MERS-CoV and SARS-CoV viruses, the SARS-CoV-2 is highly infectious and aggressive, as evident from its exponential spread, and it is shown to have a higher affinity for the ACE-2 receptor than other coronaviruses [18, 25, 37]. While MERS-CoV presents more gastrointestinal manifestations and kidney failure, SARS-CoV mainly manifests in the lower respiratory tract (LRT), possibly due to the host surface receptor affinity and distribution difference [38]. SARS-CoV entry is also through binding ACE-2 receptor, which is predominantly found in the lower respiratory tract cells [37, 39]. On the other hand, MERS-CoV facilitates access through surface enzyme dipeptidyl peptidase 4 (DPP4), also known as CD26, which is widely expressed in the LRT, kidney, and gastrointestinal tract (GIT) [40]. Genomic sequence and structure analysis of ACE2 and its side distributions across different species suggests a potential SARS-CoV-2 transmission from animals to humans [41]. Interestingly, ACE-2 expression is shown to be less in children than adults, which could be one of the reasons that SARS-CoV-2 is not highly contagious in children so far [42, 43]. The SARS-Cov-2 infection has also been reported in other tissues, including the heart, liver, skin, kidney, intestinal tract, and adipose tissue; however, why particular populations are affected with organ-specific infection is not clear [44–49].

In addition to ACE-2 receptor, recent evidence shows that SARS-CoV-2 can also bind to other cell surface markers such as Neuropilin-1, Neuropilin-2, and CD147 (EMMPRIN, a transmembrane glycoprotein of the immunoglobulin superfamily) facilitating virus cell entry, implying there could be more cell surface molecule involved in SARS-CoV-2 entry [50–53].

## Mitochondria as an emerging target in SARS-CoV-2

Mitochondria is not only the power generator of the cell but also regulates various other physiological and pathological processes, including calcium and iron homeostasis, steroid synthesis, and apoptosis [54–56]. Although mitochondrial ATP is required to maintain cellular homeostasis, unfortunately, it is also utilized for in-host viral replication [57, 58]. It is known that many single-stranded RNA viruses alter the host mitochondrial dynamics [59–62]. SARS-CoV-2 is closely related to SARS-CoV, which interacts with the host mitochondria in manipulating immune response to evade innate immunity [60]. A recent study shows that coronaviruses SARS-CoV-1, SARS-CoV-2, and MERS-CoV have similar proteins and localization patterns, supporting the hypothesis that these conserved proteins function similarly [63]. In addition, many of the SARS-CoV-2 structural and non-structural proteins encoded by various open reading frames (ORFs) has been shown to have the potential to interact with mitochondria, which will be discussed further below.

### SARS-CoV-2 and mitochondrial interaction

Mitochondria and viral interaction is a longstanding mystery yet to be resolved, but emerging evidence suggests potential interactions between them. Evidence shows that the mitochondrial network is highly susceptible to viral manipulation to evade mitochondria-mediated innate immune response [8, 64]. Since mitochondrial function is essential for various cellular processes, including cellular defense systems, exploiting its dynamics and function by the virus is highly likely. This section discusses the potential interaction between certain SARS-CoV-2 proteins, including ORFs, with mitochondria and outlines how it possibly manipulates them to establish viral infection.

#### Structural protein ‘M’

The M protein is one of the abundantly found structural proteins in SARS-CoV 2. It is the central organizer of the virus assembly and is responsible for the membrane curvature of the protein that helps interact with other structural proteins [28]. M protein has been shown to adopt two conformations; elongated M protein, which participates in spike clusters, rigidity, and membrane curvature. The compact M protein participates in flexibility and low spike density. The homotypic interaction between the M proteins is the primary foundation for the virion envelope [65]. M proteins interact with other structural proteins such as S and N proteins in regulating the size and variability of the virion [65].

Recently, most of the SARS-CoV-2 proteins have been cloned and expressed in the human cell, and through affinity-purified mass spectrometry, SARS-CoV-2 and human protein interactions have been identified [23]. This includes structural protein M, which interacts with many host mitochondrial proteins such as acyl-CoA dehydrogenase medium chain (ACADM), mitochondrial-processing peptidase subunit beta (PMPCB), pitrilysin metallopeptidase 1 (PITRM1), mitochondrial-processing peptidase subunit alpha (PMPCA), and coenzyme Q8B (COQ8B) [23]. These interactions are critical because the virus can induce cellular apoptosis, which is accomplished by major structural proteins like M protein. This is supported by a study in SARS-CoV that showed that M protein itself when overexpressed, induces apoptosis in human pulmonary fibroblast cells [66]. For apoptosis, a mitochondrial event called the mitochondrial outer membrane permeabilization releases caspases, a type of cysteine protease that executes the programmed cell death. However, many characteristic features are still unknown about the M protein and its interactions with mitochondria. Most of the M- interacting mitochondrial proteins belong to electron transport chain (ETC) subunits, metabolic enzymes, and processing peptidase [23]. However, the consequences and significance of these interactions are yet to be determined. Therefore, increasing our understanding of the interaction between structural protein M and mitochondria could help establish the targets for a better approach in treating COVID-19.

#### Non Structural proteins Nsp2, 7, 8, 10

In addition to structural protein M, SARS-CoV-2’s non-structural proteins Nsp2, 7, 8, and 10 are also predicted to interact with mitochondria. This is based on the study employed in SARS-CoV in which multidimensional protein identification technology (MudPIT) with C-terminally tagged SARS-CoV Nsp2 identified that Nsp2 interacts with Prohibitin 1 (PHB1) and PHB2, which are inner mitochondrial membrane proteins [67, 68]. However, the exact molecular function of the PHB complex is unknown, but it is believed to possess a chaperoning role in helping to maintain mitochondrial morphology. Additionally, SARS-CoV-2’s Nsp7 is also shown to interact with NDUFAF2, an NADH dehydrogenase [ubiquinone] 1 alpha subcomplex assembly factor 2 that is required for optimal assembly of Complex-I at the mitochondrial inner membrane [23]. Whereas, Nsp8 is shown to interact with various mitochondrial ribosomal subunits such as MRPS2, MRPS5, MRPS25, MRPS27, which are part of mitochondrial translation machinery [23]. Furthermore, by employing human embryo lung fibroblast cDNA library using a yeast trap method, it has been found that SARS-CoV nsp10 protein interacts with the NADH 4L subunit and cytochrome oxidase II, resulting in reduced NADH-cytochrome activity and depolarization of the inner mitochondrial membrane, suggesting a possible role of Nsp10 in the cytopathic effect of SARS-CoV [69]. Taken together, most of the SARS-CoV-2 non-structural proteins may support viral establishment and replication by modulating the mitochondrial function.

#### Open reading frame 3a (ORF3a)

Open reading frame 3a (ORF3a) encoded protein in SARS-CoV-2 is shown to target mitochondrial ubiquitin-specific peptidase 30 (USP30), a mitochondrial deubiquitinase involved in mitochondrial homeostasis and mitophagy control [70]. SARS-CoV-2 can control mitochondrial function through USP30 to help with host immunosuppression. Like other viruses, SARS-CoV-2 can also induce the neutrophil extracellular trap (NET)osis, an inflammatory response involving mitochondrial biogenesis, mitochondrial fusion, and fission to release mitochondrial DNA (mtDNA) outside of the cell [8, 71]. The release of mtDNA into the cytoplasm triggers the innate immune response and inflammation, a well-known phenomenon that is shown to occur during SARS-CoV infection [72–74]. As mtDNA levels increase, the damage and severity of the illness can progress to multiorgan failure, which is most likely cause mortality [75]. Although a certain level of mtDNA release may be necessary to elicit an immune response, an excess release may lead to severe pathologies in COVID-19.

SARS-CoV-2’s ORF3b protein is 154 amino acids, believed to be expressed from the subgenomic RNA3. It has been shown to localize to the nucleus and then translocate to mitochondria through the typical mitochondrial-targeted amphipathic α-helix [76]. It has also been demonstrated that ORF3b inhibits IFN-1 induction, mediated by retinoic acid-induced gene 1 (RIG-1) and the mitochondrial antiviral signaling protein. Thus, it could be one of the mechanisms for SARS-CoV-2 to evade innate immunity.

#### Open reading frame 7a (ORF7a)

Alongside other open reading proteins, ORFs 7a may aid with localization of the viral genome to the mitochondria, interact directly or indirectly and favor disease progression [8, 77, 78]. ORF7a is a transmembrane protein highly conserved between SARS-CoV and 2 [8]. It has also been shown that when ORF7a and Bcl-X are overexpressed in 293T and Vero cells and when ORF7a was immunoprecipitated, they found that ORF7a pulled down Bcl-X_L_ and other prosurvival proteins such as Bcl-2, Bcl-w, Mcl-1, and A 1[78]. Further, fractionation experiments demonstrate that ORF7a is localized to mitochondria and additionally learned that the transmembrane domain of ORF7a is critical in interacting with Bcl-X_L_ and for inducing apoptosis [78]. Similarly, the C-terminal transmembrane domain of Bcl-X_L_ is essential for this interaction and apoptosis induction.

Interestingly, ORF7a does not interact with pro-apoptotic proteins such as Bax, Bak, Bad, and Bid. Although ORF7a is shown to localize majorly to Endoplasmic Reticulum (ER) and Golgi Apparatus (GA), its partial localization to mitochondria is also shown [78, 79]. Thus, the partial interaction with mitochondrial is possibly through the transmembrane domain of 7a interacting with pro-apoptotic proteins essential for apoptosis induction, which could favor SARS-CoV-2 release and subsequent infection of neighboring cells [78].

#### Open reading frame 8a (ORF8a)

ORF8a has a 68% similarity in sequence between SARS-CoV and SARS-CoV-2, whose function is unclear [8]. However, it has been shown that SARS-CoV ORF8a, when expressed in Vero cells, ORF8a is localized explicitly to mitochondria [80]. In addition, ORF8a increases mitochondrial transmembrane potential, oxygen consumption, reactive oxygen species (ROS), and apoptosis by increasing caspase-3 activity [80]. Unlike ORF3a and ORF7a, which also induce apoptosis, ORF8a enhances viral replication in cultured cells [80]. Additionally, ORF8 possesses ER import signal and interacts with many ER proteins [63], which could also cause mitochondrial stress as most of the mitochondrial proteins are synthesized in ER and transported into mitochondria. Interaction with ORF8 is also evident from the work that demonstrates ORF8 could impair INF-1 signaling when expressed in cells [81].

#### Open reading frame 9b (ORF9b)

Specifically, the open reading frame ORF9b protein of SARS-CoV bind the outer mitochondrial membrane in A549, HEK 293, and THP-1 cellular models and facilitate the degradation of Dynamin-related protein (Drp1) through ubiquitination that ultimately causes mitochondrial elongation [60]. Drp1 is a GTPase required for mitochondrial fission, an essential process by which dysfunctional mitochondria are eliminated through mitophagy [82–86]. However, the exact consequence of this mitochondrial elongation is unclear, but it is believed to alter mitochondrial dynamics, favoring viral replication. In addition, ORF9b also manipulates the mitochondrial antiviral signaling (MAVS) protein through modulation of poly (C)-binding protein 2 (PCBP2) and a E3 Ubiquitin Protein Ligase, AIP4 in a way that degrades MAVS and TNF Receptor Associated Factors (TRAF) 3 and 6, which lead to decrease in IFN mediated antiviral response [60]. Since SARS-CoV-2 also possesses ORF9b, similar interactions may be possible between SARS-CoV-2 and mitochondria. A recent study confirms that SARS-CoV-2 ORF9b localizes to the outer mitochondrial membrane, associates with TOM70 and inhibits IFN-1 response [87]. The same study clearly shows the interaction between SARS-COV-2 ORF9b and TOM70 demonstrated by co-immunoprecipitation and biotin-streptavidin affinity purification mass spectrometry approaches [87]. Further, it has also been shown that the core and C-terminal domains, but not the transmembrane and clamp domains of TOM70 are required for such interaction [87]. Interestingly, overexpression of Tom70 rescued ORF9a inhibited INF signaling. This further supports the notion that Tom70’s role in activating MAVS protein that promotes apoptosis during viral infection, which is probably altered by SARS-CoV-2 infection [88, 89].

#### Open reading frame 9c (ORF9c)

Another SARS-CoV-2 viral protein, ORF9c interacts with mitochondrial proteins such as NDUFAF1 and NDUFAB1 [8, 23]. It is known that NDUFAF1 is one of the critical players involved in the assembly of Complex I, which could be affected by the interaction of ORF9c. However, NDUFAB1 is one of the subunits of Complex I required for optimal bioenergetics [90]. The interaction of ORF9c with these mitochondrial proteins may affect the initiation of electron flow and ROS production, which is necessary for optimal cellular signaling and immune response [91, 92].

**Fig. 3:**
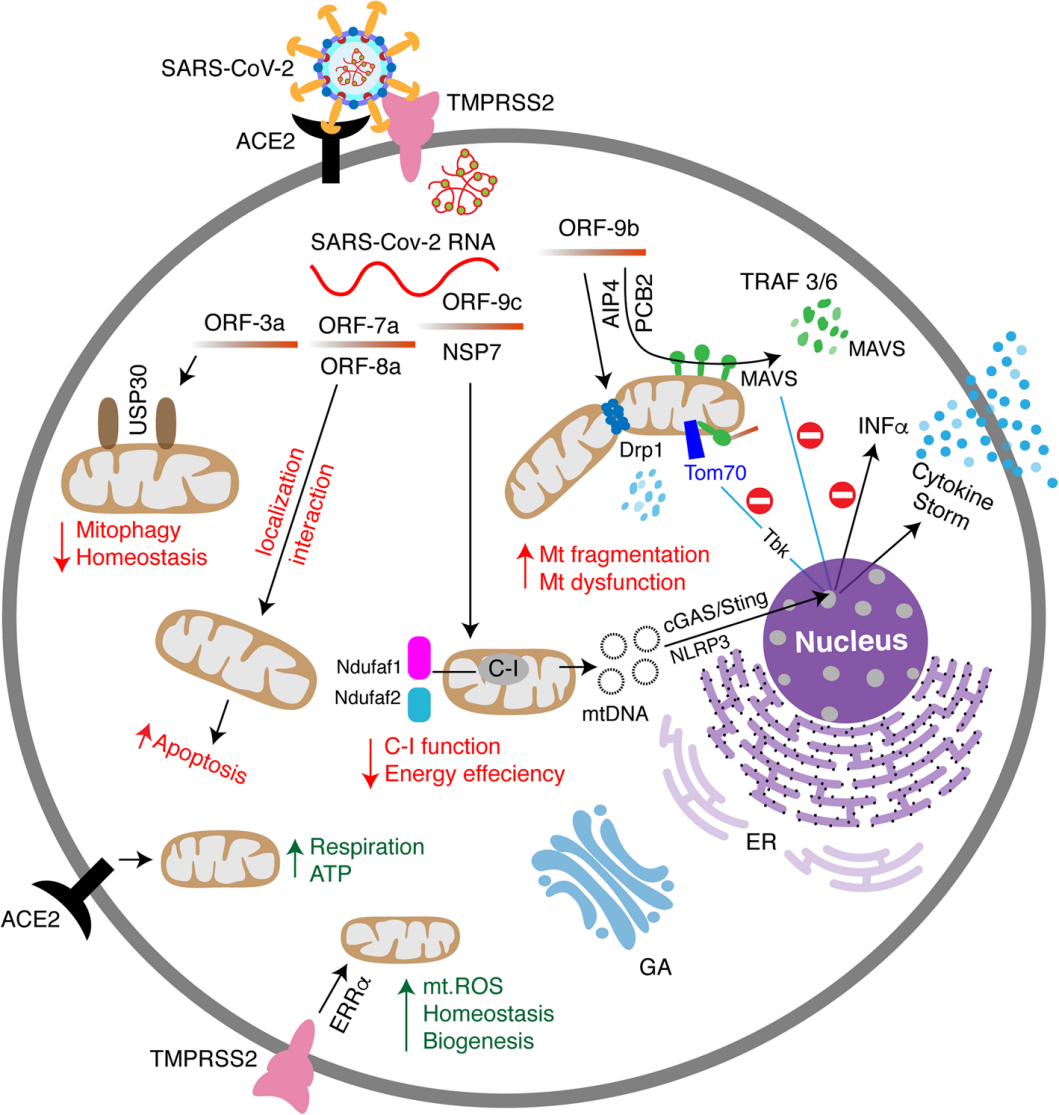
SARS-CoV-2 entry and its interaction within mitochondria. Upon SARS-CoV-2 entry, RNA genome is released, translated, and the resulting structural and non-structural proteins interact with various mitochondrial components, leading to altered host mitochondrial function. Such interactions are necessary to hijack the mitochondria so that SARS-CoV-2 escape from mitochondria-mediated innate immune response and establish its infection

So far, it is evident that SARS-CoV-2 proteins interact with mitochondrial proteins (NDUFAF1, NDUFAF2, NDUFB9, MRPS2, MRPS5, MRPS25, MRPS27) that play crucial roles in the mitochondrial metabolic pathways, required investigation to understand the mechanistic pathway of COVID-19 pathologies [23]. Other viral interactions of significance also include Tom 70, a mitochondrial importer that plays a critical role in transporting proteins into the mitochondria and, more importantly, in modulating antiviral cellular defense pathways [23, 93]. Such interactions provide viral manipulation of the host mitochondria that suppresses immunity and promotes disease progression (Fig. 3). However, a detailed and comprehensive study may require identifying the crucial mitochondrial proteins targeted by SARS-CoV-2. Preventing interactions between SARS-CoV-2 and its potential mitochondrial protein targets during virus establishment is a possible area of research in identifying potential therapeutic strategies. Recent studies have shown that MAVS is activated by the RIG-1, which can sense the presence of viral RNA [94, 95]. By interacting with viperin, an antiviral protein, MAVS can also affect interferon (IFNβ) levels, thereby acting as a means of antiviral defense [96]. Although no substantial evidence is available, it’s been proposed that SARS-CoV-2 hijack host mitochondria by all the above means to suppress immunity that aids in manipulating mitochondrial function, including the immune pathways involving the MAVS protein.

## SARS-CoV-2 manipulation of mitochondrial machinery and emergence of cytokine storm

Mitochondria are capable of altering signaling and metabolic pathways and the transcription of genes within immune cells. For example, mitochondria can switch the phenotype between pro and anti-inflammatory in immune cells [97, 98]. Furthermore, the virus relies on the mitochondria’s energy production for sustenance, which leads to the theory that modulation of mitochondrial metabolism may be effective against the virus. It is also known that replication of this virus relies on the production of double-membrane vesicles (DMVs) from the endoplasmic reticulum [99–102]. The virus replicates on these DMVs and uses them to escape the host cell’s immune defenses. Thus, it is theorized that SARS-CoV-2 manipulates mitochondria by forming double-membrane mitochondrial-derived vesicles (MDVs) [8, 103]. However, there is still no clear evidence proving MDVs promote viral replication.

Although evidence for direct manipulation of SARS-CoV-2 on mitochondria is emerging, the indirect effect of SARS-CoV-2 on mitochondria through modulating cell surface entry proteins is also possible. For example, studies have shown that ACE-2 regulates mitochondrial function [104, 105]. Also, genetic variations in ACE-2 are shown to affect mitochondrial function, as substantial variation in ACE-2 has been reported around the world, but whether such variations alter mitochondrial function and SARS-CoV-2 infection are yet to be established [106].

The cytokine storm, an extremely severe immune response, is a detrimental effect of COVID-19. The viral infection triggers inflammatory signals such as interleukins, interferons, and tumor necrosis factors during this response, leading to an excessive release of cytokines into the bloodstream. This process leads to the development of adverse side effects in the body, including tissue damage, increased cell debris known as damage-associated molecular patterns (DAMPs), and hypoxia. In addition, acute injury can ultimately lead to multiorgan failure in the most severe of cases.

As mitochondria are shown to be one of the critical components in eliciting the innate immune response, specifically in response to viruses, the SARS-CoV-2 mediated immune response involves mitochondria as well [61, 107, 108]. In support of this hypothesis, a master regulator analysis of combined SARS-CoV-2 specific interactome with MERS and SARS- CoV transcriptome using the human lung RNA-sequence data set validates not only the interaction of SARS-CoV-2 with ACE-2 and TMPRSS but also with specific mitochondrial proteins [10]. These mitochondrial proteins include MCL-1, a regulator of apoptosis, and the Complex I subunit NDUFA10 network, which are downregulated by SARS-CoV-2 [10]. However, the proposed mechanism is that the virus strategizes to down-regulate mitochondrial function and uses host cells for its replication. At normal physiological conditions, the MAVS protein present on the mitochondrion’s outer membrane interacts with mitofusin-2 (Mfn2), which is essential for mitochondrial fusion. During viral entry, associated mitochondrial membranes at the ER tether mitochondria using Mfn2 and RIG-1. Using other protein recruits, the virus binds to MAVS, which then induces phosphorylation and nuclear translocation of IRF3, resulting in the production of cytokines, including interferon I and III, through activation of NFKba and IRFs (3/7), respectively [109]. As in vitro data show that MAVS is required to induce IFN production by activating NFKβ and IRF3, recent evidence also shows that mice lacking MAVS failed to induce IFN production in response to viral infection. This suggests the possible involvement of mitochondria in the viral-mediated immune response [108] (Fig. 3).

A clue from a previous study carried out in peripheral blood mononuclear cells (PBMCs) infected with SARS-CoV shows that upregulation of genes encoding mtDNA as well as genes involved in oxidative stress, heat shock, and transcription, coincides with cytokine elevation, compared to PBMCs from control samples [110–112]. The upregulated mtDNA genes include 16S rRNA (mitochondrial ribosomal subunit), NADH dehydrogenase subunit 1 (ND1), and cytochrome c oxidase subunit I (COX1), whereas the genes involved in oxidative stress are peroxiredoxin 1 (PRDX1) and ferritin heavy polypeptide 1 (FTH1), heat shock response DnaJ (Hsp40), Dnaj homolog, subfamily B, member 1 (DNAJB1), and cytokine IL-1B as well [112]. These SARS-CoV-infected patients are also shown to have a significantly increased number of mitochondria in their PBMCs compared to PBMCs from control subjects. This may be one of the reasons why more mtDNA gene expression is observed. Interestingly, the electron microscopic structures of PBMCs from SARS-CoV infected patients show increased lysosome-like granules, which is not seen in the control. This could be due to mitochondria's activation in PBMCs, causing an increased immune response and cytokine storm. However, we hypothesize that this could be more cell-specific as it depends on whether the cell is more immunogenic or not. Likewise, a recent study has shown a similar observation of SARS-CoV-2 infection in PBMCs and lymphocytes along with the cytokine storm. However, the involvement of mitochondria, which is likely, is not investigated [113]. This study further shows that cytokines IL-6, IL-10, IL-2 and IFN-γ are significantly increased in severe COVID-19 cases than mild cases, displaying that these levels are associated with the disease severity. Therefore, a possible cytokine storm induced by mitochondria in COVID-19 could be heterogeneous among the infected population and also could be influenced by pre-existing metabolic dysfunction, which further determines the intensity of the cytokine storm and whether it eliminates the virus or causes multiorgan failure.

Identifying the critical players of sepsis and inflammatory response resulting from SARS-CoV-2 infection is essential to developing better diagnostic and therapeutic strategies. It is known that during sepsis, the infected bacteria release their DNA, which, along with the other proteins, are recognized as pathogen-associated molecular patterns (PAMPs), which stimulate the inflammatory response in the host cells. In this process, released DNA binds to the toll-like receptor 9 (TLR 9) and the formyl peptides bind to the formyl peptide receptor-1 (FPR1) on the surface of host cells. This further releases cytokines by activating p38 MAP kinase (MAPK) and attracting neutrophils while triggering immune response [114]. On the other hand, a similar pattern is also observed with no infection, but any trauma or damage to the system by external stimuli could result in the release of DAMPs, which could initiate an inflammatory response similar to PAMPs. A breakthrough study has found that mitochondria are evolutionarily conserved bacteria, sharing a similar structural motif with prokaryotes. Mitochondria could release their DNA (mtDNA) and peptides (formyl peptides), which are recognized as DAMPS similar to PAMPs, suggesting that bacteria and mitochondria use a similar tactic while eliciting an immune response. Some of the DAMPs released by the mitochondria are mtDNA, TFAM, formyl peptides and ROS [115–118]. Specifically, formyl peptides are present only in bacteria and mitochondria in nature, suggesting that the injury response caused by DAMPs is analogous to sepsis caused by bacterial infection.

Furthermore, these groups show that intravenous injection of mitochondrial DAMPs causes severe systemic inflammation, including severe lung injury, most commonly observed in COVID-19 [117, 119–121]. Also, ATP required for immune cells comes from mitochondria. In contrast, mitochondria regulate calcium buffering and ROS, which are critical components for antigen-presenting, processing and activation of signaling pathways containing inflammatory proteins [122, 123]. Specifically, in T cells, deficiency of mitochondrial transcription factor TFAM causes energy deficiency, resulting in T cell metabolic failure. This induces the circulation of cytokines, thereby establishing chronic inflammation and senescence phenotype [124]. Additionally, autophagy, a mechanism in which viruses and their proteins are eliminated by being presented to lysosomes, is shown to be impaired during mitochondrial dysfunction in SARS-CoV/CoV-2, resulting in decreased autophagy in T cells, thereby establishing the infection [125]. This is in agreement with the observation of life-threatening casualties in COVID-19 subjects with metabolic compromised pre-existing conditions such as cancer, heart diseases, diabetes, aging, obesity and chronic obstructive pulmonary disease (COPD) [126–136].

## Mitochondria’s role in SARS-CoV-2 associated comorbidities

Most of the metabolic syndromes such as diabetes, obesity, and aging are associated with mitochondrial dysfunction, which influences high-risk groups susceptible to SARS-CoV-2 infection, leading to an increase in mortality rate in these groups [131–135, 137–139]. Metabolic dysfunctions in states such as obesity and diabetes are significant players in comorbidities with COVID-19 [140–142]. This makes one speculate the importance of good mitochondrial health in resisting COVID-19 infection. Based on recent observations, COVID-19 can progress to a more severe version of respiratory disease accompanied by hyper inflammation, multiorgan failure, and death in patients with immunocompromising and pre-existing conditions such as diabetes, cardiovascular disease, and renal disease, digestive, cancer, COPD, and immunodeficiency [143]. A study also revealed an association between heart disease and COVID-19, in which congestive heart failure is one of the most common comorbidity with a 42.9% rate in patients diagnosed with COVID-19, which itself can cause heart injury [144, 145].

Interestingly, a recent study found evidence that children possess healthy mitochondria, which could protect them from severe COVID-19 pathology compared to the adult population [146]. For example, Lymphopenia is most frequently observed in the adult population infected with SARS-CoV-2 compared to children, who show milder or no symptoms of lymphopenia [146]. In this condition, T lymphocytes are reduced in adults with worsening conditions of COVID-19 when compared to children. Interestingly, it has been found that mitochondria-dependent apoptosis is elevated in T cells of adults infected with SARS-CoV-2, whereas it is not in children with COVID-19. Based on these in vitro studies, it has also been shown that increased IL-6 and TNF- α co-stimulated by ROS decreased the removal of dysfunctional mitochondria, resulting in increased mitochondrial-mediated T cell apoptosis [146]. One of the reasons could be that association of dysfunctional mitochondria with aging may cause such differences in the pathologies. Although there is a high possibility for dysfunctional mitochondrial involved in these conditions, detailed studies are required to confirm these observations.

Another theory put forward is impaired mitophagy, causing mitochondrial dysfunction in COVID-19. Mitophagy removes damaged or dysfunctional mitochondria to maintain optimal bioenergetics and cellular metabolism, further maintaining cellular homeostasis. Impaired mitophagy is one of the reasons for mitochondrial dysfunction in metabolic disorders and aging [147, 148]. In addition, viruses are believed to affect mitophagy by interfering with mitochondrial fission, fusion, and membrane potential that further causes mitochondrial stress, ROS generation and decrease ATP generation to suppress overall mitochondrial health to establish its infection [64]. Some of the viruses directly or indirectly induce mitophagy to inhibit apoptosis and prevent innate immune responses from developing their infection [149]. A recent study shows that SARS-CoV-2 interfere and inhibits autophagy by reprogramming cellular metabolism, which could be prevented by treating with autophagy inducing drugs that further prevented SARS-CoV-2 propagation [150]. Therefore, existing drugs that improve mitochondrial health could help prevent or reduce SARS-CoV-2 infection is a potential area of research.

## Potential COVID-19 treatments and role of mitochondria

**Fig. 4:**
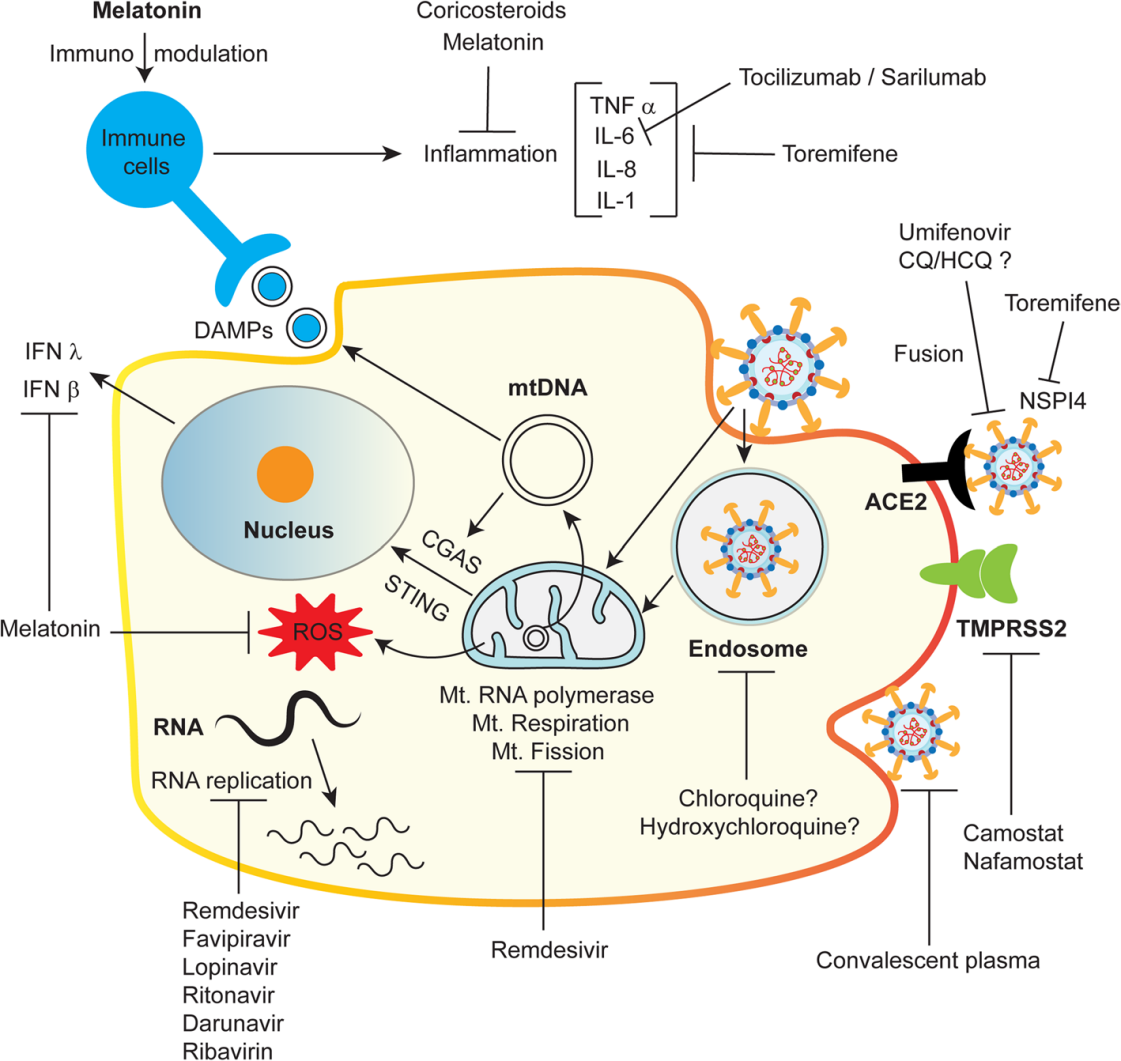
Potential proposed treatments for COVID-19. Various possible treatments for COVID-19 have been proposed since the start of the COVID-19 pandemic. Most of the proposed drugs/compounds are subjected to human clinical trials alone or combined with other drugs or strategies. Various proposed drugs include 1) adenine nucleoside analogs that target the RNA-dependent RNA polymerase, which catalyzes the replication of viral RNA (e.g., Remdesivir, Ribavirin, Favipiravir), inhibits the viral RNA replication, 2) protease inhibitors (e.g., lopinavir, ritonavir, and darunavir) that inhibits protease involved in viral replication, 3) anti-malarial drugs (e.g., chloroquine (CQ) and hydroxychloroquine (HCQ)), which are proposed to inhibit ACE-2 receptor and endocytosis, 4) a selective estrogen receptor modulator (e.g., toremifene), 5) corticosteroids (e.g., dexamethasone, hydrocortisone, prednisolone) that reduce inflammation, 6) TMPRS22 protease inhibitors (camostat and nafamostate), which prevents SARS-CoV-2 binding and entry into the cell. 7) IL-6 antibodies (tocilizumab and sarilumab) to prevent IL-mediated cytokine storm, 8) COVID-19 convalescent plasma, obtained from the COVID-19 survivors to target SARS-CoV-2 with antibodies present in the convalescent plasma, and 9) Melatonin, which has multifunctions like antioxidant, anti-inflammatory, and immune modulator

Until the recent rollout of vaccines, the prevention for COVID-19 had been unpredictable. Despite vaccine administration, people infected with SARS-CoV-2 are at high risk for developing and spreading COVID-19. Hence, treatment strategies to cure or prevent severe COVID-19 pathology or morbidity is an unmet need. Currently, a variety of compounds and targets have been investigated as potential drug molecules in treating COVID-19 (Fig. 4).

The first-ever USFDA approved drug to treat COVID-19 is remdesivir, an adenine nucleoside analog that targets the RNA-dependent RNA polymerase, which catalyzes the replication of viral RNA. Remedesivir interferes with the action of the RNA polymerization resulting in decreased viral RNA replication, shown to be effective against SARS-CoV and MERS-CoV in early studies [151]. Although remdesivir showed a superior effect against SARS-CoV-2 when compared to placebo in shortening the time to recovery in adults with SARS-CoV 2 mediated lower respiratory tract infection, remedesivir alone did not change the mortality rate, suggesting additional therapecutic approaches needed in efficetnly treating COVID-19. Interestingly, remdesivir also inhibits mtDNA-dependent RNA polymerase but at lower efficiency and inhibits mitochondrial fission mediator Drp1 [152, 153]. Also, remdesivir has been shown to produce severe adverse effects, which are probably associated with mitochondrial toxicity because mitochondrial polymerases lack the selectivity of mammalian counterparts to exclude nucleoside analogs [154, 155]. It has also been identified that remdesivir causes inhibition of mitochondrial respiration through targeting various mitochondrial proteins, including those belonging to respiratory chain complexes [156]. Inhibiting the mitochondrial respiration pathway can lead to the failure of many other significant biochemical pathways in human that could be one of the reasons limiting remdesivir’s high dose toxicity, however whether its inhibitory action of viral replication is through mitochondrial modulation not clear.

Ribavirin is another guanine nucleoside analog that prevents viral replication and translation by targeting the RNA-dependent RNA polymerase (RdRp), similar to remdesivir. A study conducted regarding the therapeutic effects of ribavirin with interferon-alpha 2b against MERS-CoV showed some improvement in patients but showed severe adverse impact on SARS-CoV patients [157, 158]. Also, in a recent study in patients with severe COVID-19, ribavirin has not improved the mortality rate [159]. Furthermore, being a nucleoside analog, ribavirin showed various toxicities, including multiorgan dysfunction, lactic acidemia, and mitochondrial toxicity [160]. Another purine nucleotide analog is a favipiravir obtained from the pro drug ribofuranosyl-5′-triphosphate or T-705, which has also been recently investigated for SARS-CoV-2 and showed viral clearance and clinical improvement within 14 days in patients with a range of mild to moderate illness. However, the study also suggested further detailed dose and duration-dependent trials for the definitive conclusion [161]. Umifenovir, which is an antiviral drug that inhibits the fusion of virus and host cell, is believed to target spike protein and host cell ACE-2 interaction. Umifenovir, when compared to lopinavir and ritonavir combined, significantly improved clinical outcomes in a recent study that suggested conducting a larger multicenter trial for umifenovir against COVID-19 [162].

Drugs such as lopinavir, ritonavir, and darunavir alone or in combination are also considered as potential drug candidates for the COVID-19 treatment. These drugs are inhibitors of protease that are crucial in processing the human immunodeficiency virus-1 (HIV-1) replication. Initially, these drug combinations were USFDA approved for HIV-1 treatment as the combination of drugs acts as antiretroviral agents. Lopinavir-ritonavir has shown past clinical studies on SARS-CoV infection, where mild improvements have been demonstrated in patients, but nothing significantly different [163]. In contrast, a clinical study on COVID- 19 patients regarding the drug efficacy showed no therapeutic benefits in hospitalized patients, but further trials in severely ill populations were recommended [164]. Cobicistat is another drug that acts as a cytochrome P450 3A isoform inhibitor, thereby increasing the absorption of co-administered drugs that act on viral replication. A clinical study showed that darunavir has no substantial antiviral activity against the SARS-CoV-2 virus [165]. Recently, a combination of darunavir/cobicistat for five days has been tested and did not show any substantial effect againt COVID-19. However, the limitation of small sample size and open-label design has been limited in this study [166].

Other potential drug candidates to treat COVID-19 under controversy are chloroquine diphosphate (CQ) and hydroxychloroquine (HCQ), which are 4-aminoquinoline class drugs, USFDA approved for the treatment of malaria and rheumatoid arthritis. Multiple clinical trials have been initiated to study the effects of these anti-malarial drugs on COVID-19 disease, and it is getting controversial as only one report showed a beneficial effect [167]. There are many proposed theories that CQs inhibit cellular entry of the virus by inhibiting SARS-CoV- 2 uptakes by ACE-2 receptor and endocytosis [168–171]. It has also been known that CQ and HCQ behave like a weak base and act through various intracellular mechanisms such as inhibiting the intracellular enzyme. It inhibits viral entry through pH-dependent endocytosis as the virus majorly relies on pH for its establishment [169, 172, 173]. These drugs are shown to targets explicitly the ACE2 receptor, which prevents the virus from entering the cell membrane by inhibiting interaction with S-protein [168]. Although in vitro and some human clinical studies show promising results, the outcomes of various human clinical trials showed no clear benefit of using these drugs to treat COVID-19 [174–176]. Also, the impact of CQs on mitochondrial function are not so intensively investigated, but it has been shown that CQ inhibits mitochondrial respiration, ATP production, and function and whether this influences the treatment is not clear [177, 178]. Also, whether its combination with other therapeutic strategies would be beneficial against COVID-19 requires further investigation.

Interestingly, toremifene, an FDA-approved anti-cancer drug that belongs to the category of a selective estrogen receptor modulator that selectively targets breast cancer, has also been shown to be a potential SARS-CoV 2 inhibitor [179, 180]. An initial study using human coronavirus host interaction and network proximity analyses of drug targets, and systems pharmacology approach identified that toremifene could be a potential drug againt SARS-CoV-2 [179]. Earlier, toremifene was shown effective against MERS-CoV and SARS-CoV [181]. Furthermore, by employing homology modeling and other bioinformatic tools, toremifene has been shown to inhibit the spike glycoprotein. In addition, a strong interaction has also been observed between toremifene and NSP14 of SARS-CoV-2, suggesting a potential therapeutic drug for COVID-19 that needs detailed investigation [180].

Other potential drugs belong to corticosteroids (e.g., dexamethasone, hydrocortisone, prednisolone), which decrease inflammatory response against influenza infections, SARS-CoV and MERS-CoV, whereas they have also been investigated against SARS-CoV-2 [182, 183]. Dexamethasone is effective in SARS-CoV-2 infected groups receiving mechanical ventilation and receiving oxygen that lowered the 28-day mortality rate, but not in groups with no kinds of support [183]. In addition, administration of methylprednisolone followed by prednisolone was also effective and showed better outcomes than the dexamethasone group against SARS-CoV-2 [184]. Out of many human clinical trials, dexamethasone is shown to be the first effective treatment that saved lives against COVID-19 [183, 185]. The primary mechanism of dexamethasone is its anti-inflammatory action through glucocorticoid receptors, which also translocate to mitochondria; however, the mitochondrial contribution to such effect needs to be explored [186].

Recently, melatonin has emerged as one of the potential enhancers/adjuvants in COVID-19 treatment [187]. Melatonin is a multifunctional molecule synthesized in mitochondria and possesses antioxidant, anti-inflammatory, and immunomodulatory functions that are shown to have a protective effect against COVID-19 [187–189]. As melatonin improves mitochondrial function by increasing oxidative phosphorylation, ATP production, upregulation of antioxidant enzymes, scavenging ROS and reactive nitrogen species (RNS), it has been proposed to test for its protective action against COVID-19 [188–194]. Although melatonin is not a standalone treatment against COVID-19, it has been suggested as a prophylactic molecule in combination with other drugs that could be useful in managing COVID-19 treatment [187].

Drugs such as camostat and nafamostate, which are TMPRS22 protease inhibitors, are also shown to be effective against SARS-CoV-2 [31, 195–197]. As TMPRS22 is responsible for SARS-CoV-2 cell entry through S protein activation, inhibiting TMPRS22 is an effective method to contain virus entry; clinical trials are underway worldwide. On the other hand, tocilizumab and sarilumab are monoclonal antibodies against IL-6 and believed to have a potential role against COVID-19 by inhibiting IL-6 mediated inflammation. However, a recent clinical trial found that these antibodies have not shown any better clinical outcomes or lower mortality rates alone or combined with remdesivir [198–201].

Altogether, based on the shreds of evidence that these drugs also influence mitochondrial function, the perspective of mitochondria as a potential target in treating COVID-19 is eminent, which is also supported by the evidence of mitochondrial manipulation by SARS-CoV-2 (Table 1). Therefore, targeting crucial interaction between SARS-CoV-2 components and mitochondrial proteins could be a potential strategy against COVID-19 (Table 1). But, many studies need to be conducted to establish mitochondria as a target in treating COVID-19. Strategies like improving mitochondrial function could by any means be detrimental to virus establishment. Therefore, treatment options can include compounds that modulate mitochondrial bioenergetic processes because the virus relies on mitochondria energy. Also, virus-induced mitochondrial dysfunction could be alleviated by increasing mitochondrial function, thereby preventing mitochondrial hijacking by the virus.

**Table 1:** SARS-CoV-2 interaction with mitochondrial function

SARS-CoV-2 Target	Interaction	Function [Reference]
Acyl-CoA Dehydrogenase Medium Chain (ACADM)	Structural protein “M’	Mitochondrial fatty acid β-oxidation (FAO) [23]
Mitochondrial-processing peptidase subunit beta (PMPCB)	Structural protein “M’	Mitochondrial precursor processing [23]
Pitrilysin metallopeptidase 1 (PITRM1)	Structural protein “M’	Mitochondrial precursor processing and degradation [23]
Mitochondrial-processing peptidase subunit alpha (PMPCA )	Structural protein “M’	Cleavage of the leader peptides of precursor proteins [23]
Coenzyme Q8B (COQ8B)	Structural protein “M’	transporting electrons along the respiratory chain of the mitochondrial inner membrane [23]
^a^Prohibitin 1(PHB1)	NSP2	Chaperone for ETC proteins [68]
^a^Prohibitin2 (PHB2)	NSP2	Chaperone for ETC proteins [68]
NADH dehydrogenase [ubiquinone] 1 alpha subcomplex assembly factor 2 (NDUFAF2)	NSP7	Chaperone for mitochondrial complex I assembly [23]
Mitochondrial ribosomal protein Subunits (MRPS2, S5, S25 and S27)	NSP8	Mitochondrial translation [23]
^a^NADH-ubiquinone oxidoreductase chain 4L (NADH4L)	NSP10	Complex I function [69]
^a^Cytochrome C oxidase subunit II (COX II)	NSP10	Complex IV function [69]
Mitochondrial ubiquitin-specific peptidase 30 (USP30)	ORF3a	Deubiquitinase [22]
^a^B-cell lymphoma-extra-large (Bcl-X_L)_	ORF7a	Apoptosis [78]
^a^Mitochondrial antiviral-signaling protein (MAVS)	ORF9b	Antiviral signaling [60]
^a^TRAF3 (TNF receptor associated factor 3),	ORF9b	Antiviral signaling [60]
^a^TRAF 6 (TNF receptor associated factor 6).	ORF9b	Antiviral signaling [60]
^a^Dynamin-related protein 1 (Drp1)	ORF9b	Mitochondrial fission [60]
Translocases of outer membrane 70, mitochondrial (Tom70)	ORF9b	Mitochondrial import [23, 87]
NADH dehydrogenase [ubiquinone] 1 alpha subcomplex assembly factor 1 (NDUFAF1)	ORF9c	Complex-I assembly factor [8, 23]
NADH: Ubiquinone Oxidoreductase Subunit AB1(NDUFAB1)	ORF9c	Complex I function [8, 23]

^a^Predicted interactions based on conserved structure and functions from SARS-CoV

## Conclusions and perspectives

The sudden outbreak and rapid spread of SARS-CoV-2 posed a severe challenge to the global welfare framework. Developing treatments and immunizations requires a thorough and profound comprehension of the pathogenesis of these arising viruses. Although we initially know little about the SARS-CoV-2, past knowledge and work on SARS-CoV and MERS-CoV sped up our understanding of this novel COVID-19 in a brief timeframe. We have put forward various hypotheses about SARS-CoV-2 pathogenesis with different investigations, related explicitly to mitochondrial interactions as SARS-CoV-2 is proposed to hijack mitochondria leading to virus establishment in the host cells. Studies show that SARS-CoV-2 uses mitochondria to escape from immunity by altering metabolic pathways and suppressing the innate immune response by interacting with various components of host mitochondria. Furthermore, the other factors related to the mitochondrial function that influence SARS-CoV-2 establishment are age, immunosuppression, and metabolic disorders (age, diabetes, obesity). There is clear evidence that the elderly population is affected with severe infection from the virus, leading to a higher mortality rate. In conclusion, understanding the intricate relationship between SARS-CoV-2 and mitochondria is critical for developing treatment options and fighting against COVID-19 and other novel RNA viruses.

## Data Availability

Not applicable

## Abbreviations

ACADM: Acyl-CoA Dehydrogenase Medium Chain
ACE-2: Angiotensin-converting enzyme-2
Bcl-X_L_: B-cell lymphoma-extra-large
COPD: Chronic obstructive pulmonary disease
COQ8B: Coenzyme Q8B
PHB1: Prohibitin 1
COVID-19: Coronavirus disease 19
COX II: Cytochrome c oxidase subunit II
COX I: Cytochrome c oxidase subunit I
DMVs: Double-membrane vesicles
DNAJB1: DNAJ homolog, subfamily B, member 1
DPP4: Dipeptidyl peptidase 4
Drp1: Dynamin-related protein 1
ERRα: Estrogen-related receptor alpha
FPR1: Formyl peptide receptor-1
FTH1: Ferritin heavy polypeptide 1
GTT: Gastrointestinal tract
HCQ: Hydroxychloroquine
MAPK: Mitogen-activated protein kinase
MAVS: Mitochondrial antiviral signaling
MDVs: Mitochondrial-derived vesicles
MERS-CoV: Middle East respiratory syndrome-CoV
ND1: NADH dehydrogenase subunit 1
NDUFAF1: NADH dehydrogenase [ubiquinone] 1 alpha subcomplex assembly factor 1
NDUFAB1: NADH: Ubiquinone Oxidoreductase Subunit AB1
NDUFAF2: NADH dehydrogenase [ubiquinone] 1 alpha subcomplex assembly factor 2
NADH4L: NADH-ubiquinone oxidoreductase chain 4L
NET: Neutrophil extracellular trap
PAMPs: Pathogen-associated molecular patterns
PCBP2: Poly (C)-binding protein 2
PHB2: Prohibitin2
PITRM1: Pitrilysin metallopeptidase 1
PMPCA: Mitochondrial-processing peptidase subunit alpha
PMPCB: Mitochondrial-processing peptidase subunit beta
PRDX1: Peroxiredoxin 1
SARS-CoV-2: Severe Acute Respiratory Syndrome-Corona Virus 2
TLR: Toll-like receptor 9
Tom70: Translocases of outer membrane 70, mitochondrial
TRAF: TNF Receptor Associated Factor
TRAF3: TNF receptor associated factor 3
TRAF6: TNF receptor associated factor 6
USP30: Ubiquitin specific peptidase 30
